# Neuroprotection by Ozanimod Following Intracerebral Hemorrhage in Mice

**DOI:** 10.3389/fnmol.2022.927150

**Published:** 2022-06-15

**Authors:** Fei Wang, Xiangyu Zhang, Yang Liu, Zhe Li, Ruixue Wei, Yan Zhang, Ruiyi Zhang, Suliman Khan, V. Wee Yong, Mengzhou Xue

**Affiliations:** ^1^Department of Cerebrovascular Diseases, The Second Affiliated Hospital of Zhengzhou University, Zhengzhou, China; ^2^Academy of Medical Science, Zhengzhou University, Zhengzhou, China; ^3^Department of Clinical Neurosciences, The Hotchkiss Brain Institute, University of Calgary, Calgary, AB, Canada

**Keywords:** intracerebral hemorrhage, Ozanimod, blood-brain barrier, brain edema, neuroprotection

## Abstract

The destruction of the blood-brain barrier (BBB) after intracerebral hemorrhage (ICH) is associated with poor prognosis. Modulation of sphingosine 1-phosphate receptor (S1PR) may improve outcomes from ICH. Ozanimod (RPC-1063) is a newly developed S1PR regulator which can selectively modulate type 1/5 sphingosine receptors. Here, we studied the impact of Ozanimod on neuroprotection in an experimental mouse model of ICH, induced by injecting collagenase type VII into the basal ganglia. Ozanimod was administered by gavage 2 h after surgery and once a day thereafter until sacrifice. The results demonstrate that Ozanimod treatment improved neurobehavioral deficits in mice and decreased weight loss after ICH. Ozanimod significantly reduced the density of activated microglia and infiltrated neutrophils in the perihematoma region. Furthermore, Ozanimod reduced hematoma volume and water content of the ICH brain. The results of TUNEL staining indicate that Ozanimod mitigated brain cell death. The quantitative data of Evans blue (EB) staining showed that Ozanimod reduced EB dye leakage. Overall, Ozanimod reduces the destruction of the BBB and exert neuroprotective roles following ICH in mice.

## Introduction

Intracerebral hemorrhage (ICH) refers to primary non-traumatic hemorrhage in the brain parenchyma. It is a severe neurological disease, with high mortality and morbidity. Globally, more than 5 million ICH patients are reported each year, where approximately 3 million people die from the insult (Krishnamurthi et al., 2013). Following the onset of ICH, only 12–39% of patients regain independent living and daily functioning (Van Asch et al., 2010). Although the administration of recombinant activated factor VIIa and management of blood pressure can attenuate hematoma and improve functional outcomes (Mayer et al., 2005; Toyoda et al., 2019), effective treatments for ICH are yet to be developed (Keep et al., 2012; Chen et al., 2021; Zhang R. et al., 2021; Zhang et al., 2022a).

Microglia are the resident immune cells in the central nervous system (CNS) (Lyu et al., 2021), with immune activities similar to peripheral immune cells such as neutrophils, lymphocytes, and monocytes (Zhao et al., 2017; Bai et al., 2020; Xue and Yong, 2020). Microglia are the first responders upon pathological changes to homeostasis after ICH (Sun et al., 2021). It has been established that disruption of the adhesions between endothelial cells (ECs) and degradation of the extracellular matrix causes irreversible blood-brain barrier (BBB) disruption. The latter leads to deteriorated pathophysiological conditions including brain edema, because of extravasation of red blood cells and the infiltration of circulatory immune cells (Zhang Y. et al., 2021; Zhang et al., 2022b). Thus, it is beneficial to reduce brain edema (Volbers et al., 2018) and restore the function of the BBB in patients with ICH. In this regard, targeting inflammation may represent a valuable approach to preserving the integrity of the BBB.

Ozanimod is a potent sphingosine 1-phosphate (S1P) 1/5 receptor agonist. In rodent studies, Ozanimod showed a neuroprotective effect on CNS diseases (Cohan et al., 2020). The selectivity of Ozanimod for S1PR1 is 27 times that for S1PR5, and the selectivity for S1PR1 is more than 10,000 times that for S1PR2, 3, and 4 (Scott et al., 2016). The S1PR1 subtype is present in cells of the nervous system, including astrocytes, oligodendrocytes, and neural progenitors, where it affects neurogenesis, cell migration, neurotransmission, and survival (Sun et al., 2016). Its expression on endothelial cells affects endothelial barrier function, and it co-regulates BBB function with its expression on astrocytes. The S1PR5 subtype is mainly expressed in oligodendrocytes of the central nervous system and mediates proliferation, differentiation, cell-cell interactions of the oligodendrocyte lineage, as well as neuroprotection (Van Doorn et al., 2012; Li et al., 2020). The high selectivity of Ozanimod for S1PR1 minimizes potential safety issues related to the activation of S1PR3 because there are many adverse events associated with SIPR3 (and other S1PR subtypes) which include hypertension, macular edema, pulmonary toxicity, and liver toxicity (Cohen and Chun, 2011; Gold et al., 2014; Cohen et al., 2016). Currently, the effects of a modulator of the S1PR1/5 have not been studied in ICH. In this study, we investigated the therapeutic benefits of an oral S1PR1/5 modulator Ozanimod in a mouse model of ICH.

## Materials and Methods

### Animals

120 adult male C57BL/6J mice (8–10 weeks old) were provided by Beijing Vital River Experimental Animals Centre (Beijing, China). This study was conducted in accordance with the Animal Care Committee of Zhengzhou University. All animals were maintained in a specified pathogen-free (SPF) environment, with temperature of 25°C, relative humidity of 55%, a light/dark cycle of 12 h, and free access to food and water.

### Intracerebral Hemorrhage Models

The collagenase-induced ICH model was established as described previously (Xue et al., 2006; Liu et al., 2021). In brief, the mice were anesthetized with pentobarbital (40 mg/kg) by intraperitoneal injection. After successful anesthesia, the mice were placed on a stereotaxic instrument (RWD, Shenzhen, China). A small incision was made on the skin after shaving and alcohol cleaning, and the bregma was exposed by separating the subcutaneous tissue layer by layer to the surface of the skull. The skull plane was then adjusted to make the bregma and lambda at equal level. A round hole with a diameter of 0.6 mm was drilled into the skull, located 0.8 mm in front of the bregma and 2.0 mm on the right side of the midline, reaching the surface of the dura mater. Collagenase solution (0.075U in 0.5μL saline, VII; Sigma-Aldrich, Milwaukee, WI, United States) was infused at a rate of 0.1 μL/min using a Hamilton syringe under a depth of 3.5 mm below the surface of the skull, after which the needle was left for 10 min to prevent backflow. Subsequently, the incision was sutured and disinfected using iodophor. The sham operation group was injected with the same volume of saline into an identical intracranial location as collagenase, and the other steps were also identical to those of the ICH mice. Animals were strictly monitored until fully recovered from anesthesia.

### Study Design and Drug Treatment

**FIGURE 1:**
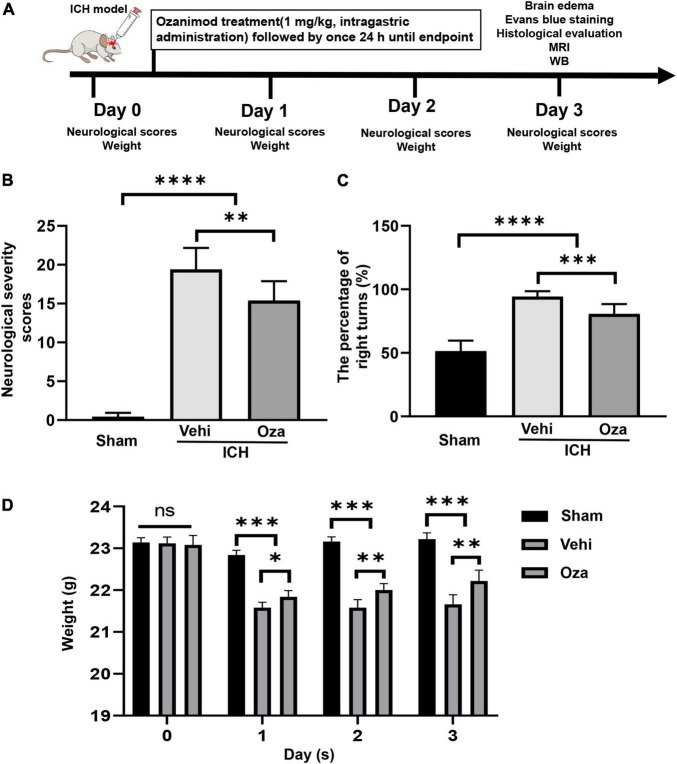
The effects of Ozanimod on neurological function and weight changes in mice after ICH. **(A)** Flow chart of animal experiments. **(B,C)** Ozanimod improved functional results on the neurological severity scores and corner turn test (*n* = 6). **(D)** Ozanimod reduced postoperative weight loss in mice (*n* = 5). Data are presented as mean ± *SD*. *p < 0.05, ^**^p < 0.01, ^***^p < 0.001, ^****^p < 0.0001.

All animal studies were performed according to guidelines of ARRIVE (Animal Research: Reporting of *in vivo* Experiments) (Kilkenny et al., 2010; Percie Du Sert et al., 2020). A total of 120 adult C57BL/6 male mice (8–10 weeks) were randomly assigned into three groups: the sham group, the vehicle (ICH + DMSO) group, and the treatment (ICH + Ozanimod) group. The brains of all mice were collected at 3 days after ICH. We chose the 3-day time point because our previous studies demonstrated that brain edema, impairment of the BBB and the severity of the neurological deficit peaked on the 3rd day of ICH in mice (Xue et al., 2010). For the treatment settings, Ozanimod (Topscience, Shanghai, China) was dissolved in 0.5% dimethyl sulfoxide (DMSO) and diluted with 0.9% saline. After pre-testing with 0.3 mg/kg and 1 mg/kg Ozanimod, we learned that the optimal drug concentration was 1 mg/kg in ICH mice (Scott et al., 2016). The treatment group was given intragastric administration of Ozanimod (1mg/kg) once a day, and sham/vehicle group was treated with an equivalent amount of 0.5% DMSO once a day. Data were collected and analyzed by two blinded investigators. The flow chart of this experiment is showed in Figure 1A.

### Neurological Function Tests

Corner turn test: The mice were placed in a corner made of plexiglass with an angle of 30°. The mice spontaneously moved forward until the whiskers touched the plexiglass corner. The turning direction was recorded and all animals were observed 20 times in succession, with each interval not less than 1 min. The percentage of right turns [right/(right + left)] was calculated.

Neurological severity scores: In this experiment, the tests included body symmetry, gait, climbing, circling behavior, front limb symmetry, and compulsory circling. Each test was assigned a range of scores from zero to four, and the total score of 24 was regarded as the most serious defect.

### Hematoma Volume Assessment

Mice were anesthetized deeply with pentobarbital (40 mg/kg). After perfusion, the brain was immediately taken out and fixed with 4% phosphate-buffered paraformaldehyde. The fixed brain was cut from the frontal lobe to the occipital lobe with a thickness of 1 mm. The brain slices were arranged in order. We used Image-Pro + 5.0 software and the formula V = t (A_1_ + A_2_ + ⋅⋅⋅ + A_n_), where V is the volume of the hematoma, t is the slice thickness, and A is the area of the hematoma to measure and calculate the volume of hematoma (cubic millimeters) (Song et al., 2003; Fang et al., 2014).

### MRI Scanning and Measurements

We used small animal MRI system (MRS-4717, MR Solution company) to measure the water content of brain after ICH. The magnet type is superconducting, and the external environment does not affect the image quality. The main magnetic field strength is 4.7 T. The diameter of the magnet is 170 mm, and the highest magnetic field uniformity is 0.05 ppm/h. The cooling type is a dry magnet to avoid consumption of liquid helium, and the gradient inner diameter is 100 mm. The mice were anesthetized with 2% isoflurane during MRI, with the following parameters: (FSE, T2w; TR, 5,000 ms; TE, 51 ms; Field, 4.7 T; Flip Angle, 90°; Slice Thk, 0.8 mm; DFOA, 25.6 mm; Zoom, 221%). The calculation method of the volume of the lesion: Combine the lesion area of the T2 phase and multiply it by the slice thickness to obtain the total volume.

### Brain Parenchymal Water Content Assessment

Brain parenchymal water content was measured on day 3 after ICH. The animals were anesthetized with pentobarbital (40 mg/kg) and then the brains were extracted quickly. After that, the brains were divided into ipsilateral, contralateral cerebral hemisphere, and the cerebellum (for internal reference). Brain samples were weighed on a precision electronic balance immediately after collection to determine wet weight. Then the weighed brain tissue was dried in the oven at 90°C for 24 h. After drying, it was taken out and its dry weight was measured again. The water content of the brain was calculated as [(wet weight—dry weight)/wet weight] × 100%.

### Immunohistochemistry Staining

The mice were anesthetized with an overdose of pentobarbital (40 mg/kg), and the heart was perfused with 0.1 mol/L PBS (pH = 7.4) containing 4% paraformaldehyde. The brain was cut into 5-μm-thick coronal sections for immunohistochemical staining. In brief, the sections were incubated with the primary antibodies overnight at 4°C, including rabbit anti-myeloperoxidase (MPO) monoclonal antibody (neutrophils marker, 1:800, Abcam, Cambridge, MA, United States), and rabbit anti-Iba1 polyclonal antibody (microglia marker, 1:200, Wako, Japan). The sections were washed with PBS (pH = 7.4) 3 times, 3 min each time. Sections were then incubated with horseradish peroxidase (HRP) combined with the secondary antibody rabbit immunoglobulin G (IgG) antibody (Servicebio, Wuhan, China) for 1 h at room temperature.

### Immunofluorescent Staining

After the mice were perfused, brain tissue samples were fixed in phosphate buffered 4% paraformaldehyde at 4°C overnight. The mouse brain was then cut into 5-μm thick coronal sections for immunofluorescent staining. Slices were incubated with primary antibodies including mouse anti-ZO-1 monoclonal antibody (1:300, Abcam, Cambridge, MA, United States) and mouse anti-Occludin monoclonal antibody (1:100, Abcam, Cambridge, MA, United States) overnight at 4°C. Then, the sections were washed 3 times with PBS (pH = 7.4), and were incubated with a secondary antibody (Alexa Fluor 488 conjugated goat anti-mouse, Abcam, Cambridge, MA, United States) for 1 h at room temperature. Finally, sections were washed with PBS and stained with DAPI. All sections were visualized and photographed by using a fluorescence microscope (Olympus Co., Japan).

### Immunofluorescent Double Staining (TUNEL and NeuN)

As in previous experiments (Zhang et al., 2022d), TUNEL staining was performed according to the manufacturer’s instructions (Vazyme Biotech, Nanjing, China). In brief, the sections were performed with 10 μg/mL proteinase K for 15 min at 37°C. After a 5-min wash in PBS, the labeled solution (labeled nucleotides and TdT enzyme) was incubated in the dark for 60 min at 37°C. The slides were then incubated with rabbit anti-NeuN antibody (1:600, Abcam, Cambridge, MA, United States) overnight at 4°C. After probing with secondary antibody (Alexa Fluor 488 conjugated goat anti-rabbit) (Abcam, Cambridge, MA, United States), the sections were counterstained with DAPI for 10 min. TUNEL and NeuN-positive cells were counted as dead neurons.

### Western Blot Analysis

The mice were euthanized with pentobarbital (40 mg/kg) at 3 days after ICH, and brain samples were taken from the 1 to 1.5 mm area around the hematoma. Brain tissues were stored at –80°C until analysis. In short, brain tissues were lysed with RIPA lysis buffer (Solarbio, Beijing, China) for 10 min, then were homogenized at 4°C and centrifuged at 12,000 rpm for 10 min before the supernatant was collected for subsequent experiments. The supernatant was collected and the protein concentration was determined with the BCA protein assay Kit (Beyotime, China). All operations were performed on ice. Equal amounts of protein were loaded on an sodium dodecyl sulfate-polyacrylamide gel electrophoresis (SDS-PAGE), electrophoresed, and then transferred to polyvinylidene difluoride (PVDF) membranes. The primary antibodies used in this study were rabbit anti-ZO-1 monoclonal antibody (1:5,000, Abcam, Cambridge, MA, United States) and rabbit anti-Occludin monoclonal antibody (1:1,000, Abcam, Cambridge, MA, United States). After incubating with primary antibodies, the membrane was washed with TBST 3 times for 15 min each. Then HRP-conjugated anti-rabbit secondary antibody IgG antibody (Servicebio, Wuhan, China) was applied at room temperature for 1 h. Finally, an enhanced chemiluminescence (ECL) detection kit was used for the visualization of bands. Image J was used to analyze the optical density (OD) of the signal.

### Evans Blue Staining

The integrity of the BBB was evaluated by measuring the amount of exudation of Evans blue (EB). Evans blue dye (prepared with normal saline, 2% concentration, 4 ml/kg) was injected through the tail vein at 3 days after ICH in mice. After 2 h, the heart of the mouse was perfused with 0.1 mol/L phosphate buffered saline (PBS) to wash away the remaining dye in the blood vessels. The brain tissues of each group were quickly collected. The tissues immersed in 4 ml methanamide solution were placed in a water bath with a constant temperature of 55°C for 24 h. The absorbance of the supernatant was measured with a spectrophotometer at 610 nm. The content of Evans blue present in the brain tissue was measured quantitatively according to a linear standard curve. The experimental results were expressed as the ratio of the EB content in the ipsilateral/contralateral brain.

### Statistical Analysis

All data were expressed as mean ± standard deviation. All analyses were blinded. Analysis of variance and Bonferroni *post-hoc* test were used for comparison between multiple groups. The *t*-test was used for analysis of 2 groups. The difference of *p* < 0.05 indicates statistical significance. Statistical analyses were performed using GraphPad Prism 8.0.1 software.

## Results

### Ozanimod Promotes Recovery of Neurological Function and Body Weight After Intracerebral Hemorrhage

Ozanimod treatment markedly restored neurological function in the ICH model. Neurological severity scores of the Ozanimod group were significantly lower than those in the ICH + Vehicle group (Figure 1B, *p* < 0.01). We analyzed behavioral performance using the corner turn test, which showed that compared with the ICH + Vehicle group, the number of right turns was significantly reduced in the Ozanimod group (Figure 1C, *p* < 0.001). During the experiment, we measured the weight changes of mice before and after the operation. Through statistical analysis, we found that the weight loss of mice with experimental ICH was reduced after drug intervention (Figure 1D, *p* < 0.01).

### Ozanimod Reduces Hematoma Volume and Brain Edema After Intracerebral Hemorrhage

**FIGURE 2:**
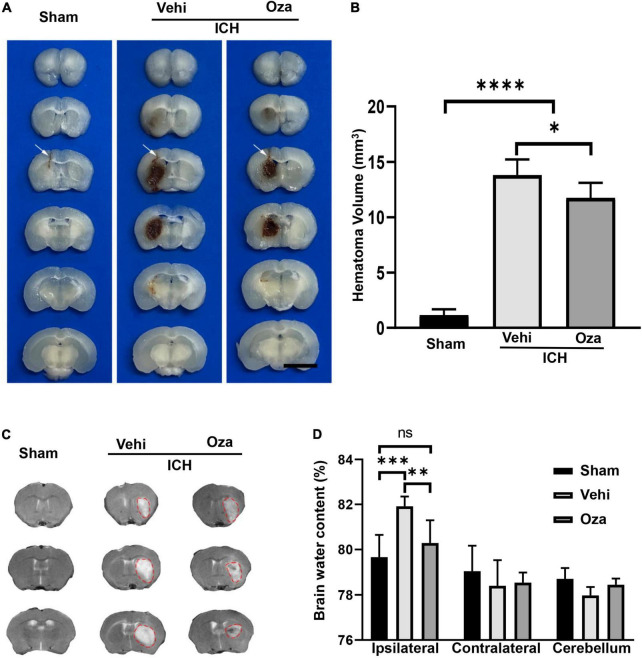
The effects of Ozanimod on hematoma volume and brain edema in mice at 3 days after ICH. **(A)** The arrow shows the pinhole (Bar = 5 mm). **(B)** The volume of hematoma was reduced in the Ozanimod group compared to the vehicle group (*n* = 8). **(C)** Representative MRI images of ICH before and after Ozanimod treatment. **(D)** Ipsilateral brain water content (*n* = 6). Data are presented as mean ± *SD*. *p < 0.05, ^**^p < 0.01, ^***^p < 0.001, ^****^p < 0.0001.

The hematoma volume of ICH in experimental mice was calculated by traditional methods (Figure 2A), and it was significantly decreased in the Ozanimod group as compared with the vehicle group at 3 days post-ICH (Figure 2B, *p* < 0.05). The results of MRI (Figure 2C) evaluation and wet/dry method also showed that brain edema in the Ozanimod group was significantly reduced at 3 days after ICH (Figure 2D, *p* < 0.01).

### Ozanimod Alleviates Neuroinflammation After Intracerebral Hemorrhage

**FIGURE 3:**
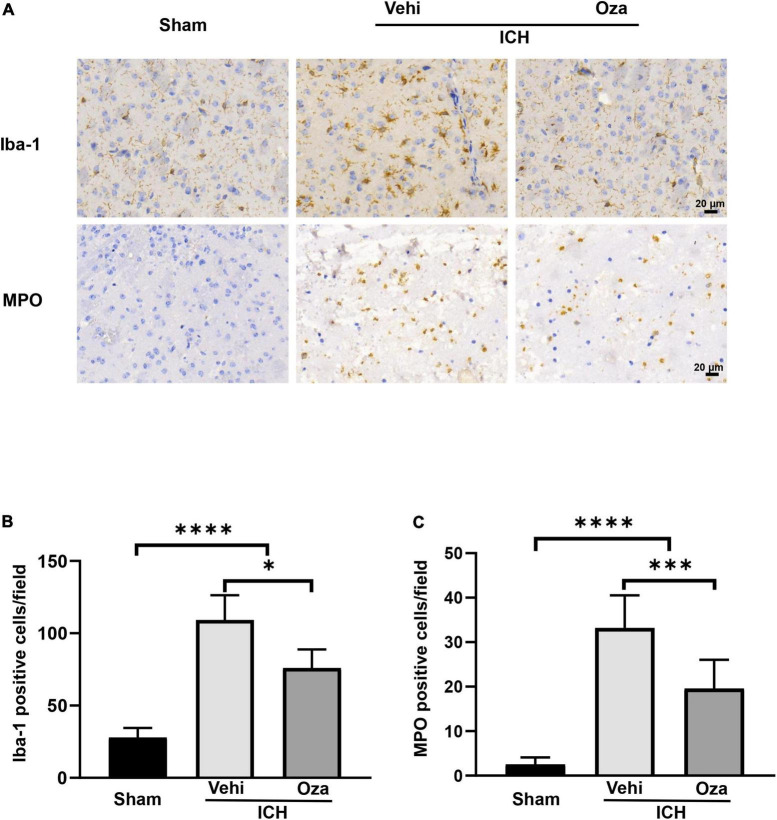
The effects of Ozanimod on microglia/macrophages density and neutrophil infiltration at 3 days after ICH. **(A)** Immunohistochemical results showed microglia (Iba-1) representation and neutrophil (MPO) infiltration around the hematoma. **(B,C)** A significant reduction in infiltration of neuroinflammatory cells was found in the Ozanimod compared to the vehicle group. Data are presented as mean ± *SD* (*n* = 6, Bar = 20 μm). *p < 0.05, ^***^p < 0.001, ^****^p < 0.0001.

Microglia are considered to be the first responders to insults in the brain. By immunohistochemical staining (Figure 3A), we found that the number of microglia was significantly reduced in the perihematoma region in the Ozanimod group after 3 days of ICH in mice; there was also less infiltrated neutrophils (Figure 3B, *p* < 0.05; Figure 3C, *p* < 0.001). These results indicate that ozanimod treatment reduced post ICH neuroinflammation.

### Ozanimod Reduces Intracerebral Hemorrhage -Induced Neuronal Death After Intracerebral Hemorrhage

**FIGURE 4:**
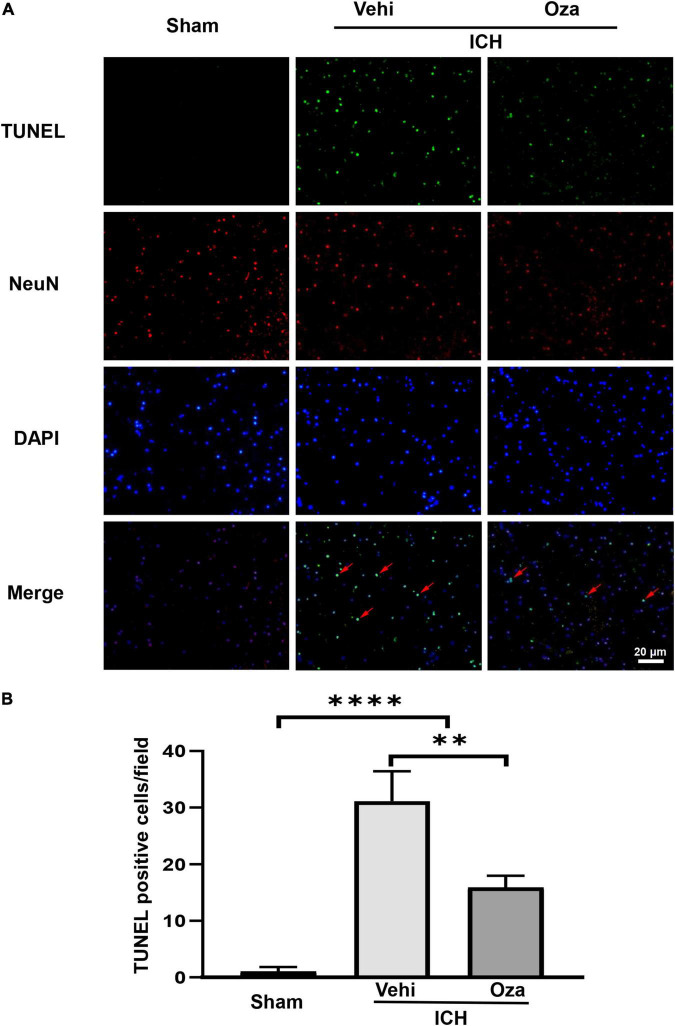
The effects of Ozanimod on neuronal death in mice after ICH. **(A)** TUNEL staining was used to mark dead cells (green). Immunofluorescence staining with NeuN was used to mark neurons (red), with the red arrow demonstrating dead neuronal cells. **(B)** Immunofluorescent staining and statistical results of death of neurons with Ozanimod or vehicle administration in mice for 3 days after ICH. Data are presented as mean ± *SD* (*n* = 6, Bar = 20 μm). ^**^p < 0.01, ^****^p < 0.0001.

The loss of neurons after ICH contributes to increased neurobehavioral scores. To evaluate neuronal death, we used TUNEL and NeuN double staining (Figure 4A), which showed that the number of dead neurons was decreased with Ozanimod treatment (Figure 4B, *p* < 0.01).

### Ozanimod Improves Blood-Brain Barrier Integrity After Intracerebral Hemorrhage

**FIGURE 5:**
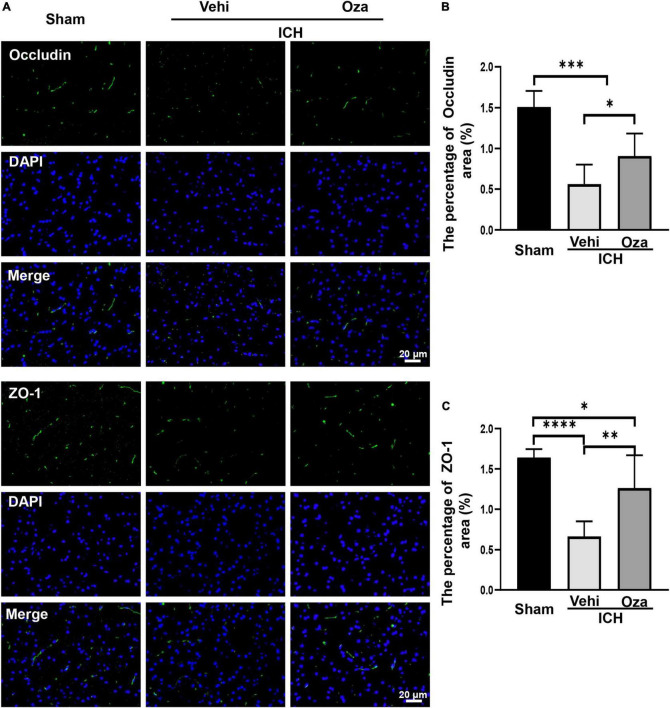
The effects of Ozanimod on inhibition of the expression of Occludin and ZO-1 at 3 days after ICH. **(A)** Immunofluorescence staining (green) of tight junction associated proteins (Occludin and ZO-1). DAPI (blue) was used to label nucleus. **(B,C)** Statistical analysis of positive area of Occludin and ZO-1 protein. Data are presented as mean ± *SD* (*n* = 6). Bar = 20 μm. *p < 0.05, ^**^p < 0.01, ^***^p < 0.001, ^****^p < 0.0001.

**FIGURE 6:**
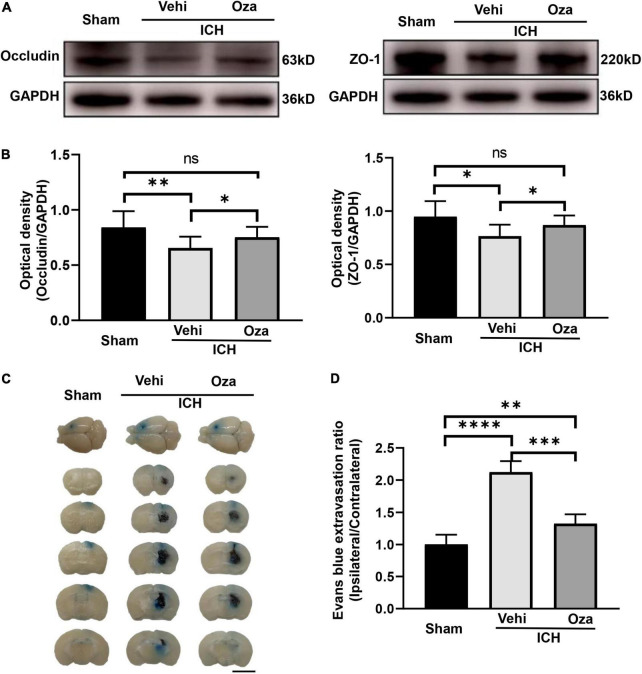
The effects of Ozanimod on blood–brain barrier permeability in mice after ICH. **(A)** Representative Western blotting images of ZO-1 and Occludin expression with and without Ozanimod treatment. **(B)** Quantitative analysis of ZO-1 and Occludin protein expression (*n* = 3). **(C)** EB dye extravasation into tissue is apparent in coronal cross section images (Bar = 5 mm). **(D)** Statistical analysis of EB exudation (*n* = 6). Data are presented as mean ± *SD*. *p < 0.05, ^**^p < 0.01, ^***^p < 0.001, ^****^p < 0.0001.

Ozanimod contributed to the maintenance of BBB integrity by increasing the tight junction associated proteins ZO-1 and Occludin in the damaged brain tissue. By immunofluorescent staining (Figure 5A), we found that the signal intensity of ZO-1 and Occludin in the ICH + Ozanimod group was significantly higher than that in the ICH + Vehicle group (Figure 5B, *p* < 0.05; Figure 5C, *p* < 0.01). The western blot data also demonstrated that protein expressions of ZO-1 and Occludin was markedly improved after the administration of Ozanimod (Figure 6A, *p* < 0.05; Figure 6B, *p* < 0.05). In addition, EB staining was used to assess BBB disruption (Figure 6C). We found that EB extravasation was significantly decreased after the intervention of Ozanimod (Figure 6D, *p* < 0.001), indicating that Ozanimod improves the integrity of BBB after ICH.

## Discussion

Ozanimod, a small molecule modulator of S1PR1/5, has shown efficacy in clinical trials in relapsing multiple sclerosis and ulcerative colitis (Sandborn et al., 2016; Lamb, 2020). Ozanimod was reported to exert anti-inflammatory effects in these trials. In the present study, Ozanimod significantly decreased inflammatory cells, protected BBB, reduced the degree of brain edema and improved neurological function in mice after ICH.

Although the mechanism underlying ozanimod mediated regulation of BBB integrity is not entirely clear, S1PR1 signaling may promote BBB integrity in the mouse brain (Yanagida et al., 2017). Also, continuous activation of S1PR1 may cause actin formation in ECs (Spindler et al., 2010), which can directly promote normal maintenance of tight junction associated protein complexes. Earlier studies have shown that specific knockdown of S1PR1 after birth induces loss of vascular endothelial growth factor (VEGF) and vascular endothelial growth factor 2 (VEGFR2) at cell junctions (Gaengel et al., 2012). Moreover, as a key regulator of neovascularization, S1PR1 plays a key role in BBB development (Jung et al., 2012; Shao et al., 2015). Studies have also shown that S1PR5 regulates the tight connection of BBB in brain ECs (Van Doorn et al., 2012; Spampinato et al., 2021). Our current study indicates that ozanimod exerts protective effect on the BBB, however, further investigations are required to explore the associated molecular mechanisms.

Ozanimod has an advantage as a selective functional antagonist of S1PR1/5 subtypes in experimental ICH, with a short half-life, and less off-target effects on blood pressure and heart rate. We further found that ozanimod reduced brain edema in experimental ICH in accordance with the recently reported positive role of selective S1PR1 regulators (Sun et al., 2016; Bobinger et al., 2019). Siponimod, for example, has shown a tendency to reduce intracerebral edema. Experimental intracerebral edema is a prominent cause for the increase in neurobehavioral scores in mice (Durocher et al., 2021).

After intracerebral hemorrhage, neuronal cell death is an important driver of neurological deficits (Zhang et al., 2022c). Therefore, inhibiting neuronal cell death after intracerebral hemorrhage can help alleviate its clinical manifestations. In this study, we detected dead neurons around the bleed by labeling NeuN-positve neurons with TUNEL. Importantly, Ozanimod intervention significantly improved neuronal survival. The specific mechanism remains to be elucidated.

BBB disruption promotes transendothelial migration of neutrophils and activation of microglia, which are major steps in neuroinflammation. Inflammation is a complex immune response after injury, and neuroinflammation is one of the key factors determining the prognosis of neurological diseases. Although FTY-720, also known as Fingolimod, reduces neuroinflammation, several side effects have been reported (Shim and Wong, 2016) and it has no protective effect on BBB functions (Schuhmann et al., 2015). In contrast, Ozanimod reduced neuroinflammation (Musella et al., 2020) and maintained BBB function in experimental ICH mice. Relevant studies have also confirmed that tight junction associated protein plays a significant role in the maturation of the BBB and the recovery of neural function (Yang et al., 2021). In addition, immunosuppression and infection caused by stroke are the main causes of death in stroke patients (Shim and Wong, 2016). FTY-720 has a long half-life, which may be one of the reasons for its toxicity (Comi et al., 2010; Scott et al., 2016). Ozanimod has a significantly shorter half-life than FTY-720 and only acts on two of the five receptor subtypes that are mostly expressed on immune cells (Derfuss et al., 2020); this may avoid the adverse side effects of immune regulation.

Our study is limited by several factors. We only employed the collagenase type VII-induced ICH model; more models including the autologous blood-initiated paradigm should be included because collagenase is a kind of foreign matter that has side effects, such as inducing an inflammatory response. The result of Ozanimod protection would be more compelling if other models of ICH are used in future studies (Liddle et al., 2020). Finally, considering that the prognosis of ICH is determined by a variety of factors, drug intervention may not be sufficient on its own to improve prognosis. Therefore, further experiments are needed to investigate these issues.

## Conclusion

We demonstrate for the first time that Ozanimod significantly enhances the integrity of the BBB in a mouse model of ICH. We also found that Ozanimod reduces neuroinflammation and improves neurobehavioral recovery following ICH in mice.

## Data Availability Statement

The raw data supporting the conclusions of this article will be made available by the authors, without undue reservation.

## Ethics Statement

The animal study was reviewed and approved by the Ethics Committee of the Second Affiliated Hospital of Zhengzhou University.

## Author Contributions

FW wrote the manuscript and analyzed the data. XZ, YL, ZL, RZ, RW, YZ, and SK performed the experiments and collected the data. VY consulted and edited the manuscript. MX supervised the project. All authors discussed the results and agreed on the content of the manuscript.

## Conflict of Interest

The authors declare that the research was conducted in the absence of any commercial or financial relationships that could be construed as a potential conflict of interest.

## Publisher’s Note

All claims expressed in this article are solely those of the authors and do not necessarily represent those of their affiliated organizations, or those of the publisher, the editors and the reviewers. Any product that may be evaluated in this article, or claim that may be made by its manufacturer, is not guaranteed or endorsed by the publisher.

## Abbreviations

S1PR: Sphingosine 1-phosphate receptor
ICH: Intracerebral hemorrhage
BBB: Blood-brain Barrier
ECs: Endothelial cells
CNS: Central nervous system.
